# Metabolic profiling reveals altered amino acid and fatty acid metabolism in children with Williams Syndrome

**DOI:** 10.1038/s41598-024-83146-4

**Published:** 2024-12-28

**Authors:** Weijun Chen, Yang Yang, Yu Zhang, Changxuan Sun, Chai Ji, Jiyang Shen, Fangfang Li, Yingping Xiao, Yang Wen, Qian Liu, Chaochun Zou

**Affiliations:** 1 Department of Child Health Care, Children’s Hospital, Zhejiang University School of Medicine, National Clinical Research Center for Child Health, 3333 Binsheng Road, Hangzhou, 310052 Zhejiang Province China; 2 Yunnan Provincial Key Laboratory of Public Health and Biosafety & School of Public Health, Kunming Medical University, Kunming, 650500 People’s Republic of China; 3 R&D Department, Zhejiang Biosan Biochemical Technologies Co. Ltd, 859 Shixiang West Rd, Hangzhou, 310007 Zhejiang Province China; 4 Suzhou Dushu Lake Hospital, The Fourth Affiliated Hospital of Soochow University, Medical Center of Soochow University, Suzhou, 215123 Jiangshu Province P. R. China; 5 State Key Laboratory for Managing Biotic and Chemical Threats to the Quality and Safety of Agro-Products, Institute of Agro‐product Safety and Nutrition, Zhejiang Academy of Agricultural Sciences, Hangzhou, China; 6 Medical Department, Zhejiang Biosan Biochemical Technologies Co. Ltd, 859 Shixiang West Rd, Hangzhou, 310007 Zhejiang Province China; 7 Department of Endocrinology, Children’s Hospital, Zhejiang University School of Medicine, National Children’s Regional Medical Center, National Clinical Research Center for Child Health, 3333 Binsheng Road, Hangzhou, 310052 Zhejiang Province China

**Keywords:** Williams Syndrome, Targeted metabolomics, Long-chain saturated fatty acids (LC-SFAs), Docosahexaenoic acid (DHA), Arachidonic acid (ARA), Amino acids, Metabolomics, Neurodevelopmental disorders, Paediatric research

## Abstract

Williams Syndrome (WS) is a rare neurodevelopmental disorder with a prevalence of 1 in 7500 to 1 in 20,000 individuals, caused by a microdeletion in chromosome 7q11.23. Despite its distinctive clinical features, the underlying metabolic alterations remain largely unexplored. This study employs targeted metabolomics to investigate the metabolic characteristics of children with WS. Using liquid chromatography-tandem mass spectrometry (LC-MS/MS), we identified significant dysregulation of 15 metabolites, with 11 upregulated and 4 downregulated. Notably, amino acids such as alanine, proline, and arginine were significantly elevated. Fatty acid metabolism showed pronounced upregulation of long-chain saturated fatty acids (C18:0, C20:0, C22:0, C24:0, C26:0, and C28:0) and downregulation of long-chain unsaturated fatty acids (C18:2 LA, C22:6 DHA, C16:1 PLA, and t-C18:1 EA), except for upregulated nervonic acid (C24:1) and arachidonic acid (C20:4). Metabolic pathway analysis highlighted disruptions in arginine synthesis, arginine/proline metabolism, alanine, aspartate and glutamate metabolism, biosynthesis of unsaturated fatty acids, linoleic acid metabolism, and arachidonic acid metabolism. This study provides the first comprehensive analysis of amino acid and fatty acid metabolism in children with WS, offering insights into the disorder’s complex metabolic landscape. Further validation in larger cohorts is essential to confirm these findings and their potential as biomarkers and therapeutic targets.

**Supplementary Information:**

The online version contains supplementary material available at 10.1038/s41598-024-83146-4.

## Introduction

Williams Syndrome (WS), also known as Williams-Beuren Syndrome (OMIM#194050), is a rare genetic disorder caused by a microdeletion within the Williams-Beuren Syndrome Critical Region (WBSCR) on chromosome 7q11.23, which contains 25–27 genes^1,2^. The estimated prevalence of this neurodevelopmental disorder ranges from 1/20,000 to 1/7500^3^. WS is characterized by distinctive clinical features, including unique facial features, cardiovascular abnormalities (notably supravalvar aortic stenosis, peripheral pulmonary stenosis, and hypertension), developmental delays, mild intellectual disability, and endocrine abnormalities such as hypercalciuria and hypothyroidism, and diabetes mellitus^4–9^. Despite these well-documented clinical features, the complex metabolic alterations responsible for these manifestations have remained largely unexplored, and the underlying pathophysiological mechanism of WS is a topic worthy of further exploration. Given that metabolism plays a pivotal role in energy production, growth, and cognitive functions, exploring the metabolic pathways affected in children with Williams syndrome is critical to understanding the underlying biochemical disruptions contributing to the clinical phenotype.

Among the metabolic pathways potentially disrupted in WS, amino acid and fatty acid metabolism are of particular interest due to their pivotal roles in protein synthesis, energy homeostasis, and cell signaling. Alterations in these pathways may provide valuable insights into the metabolic disturbances that contribute to key clinical features, such as developmental delays^10–14^, cardiovascular abnormalities^12,15–19^, and neurological deficits^20–22^.While these disruptions are not unique to WS, they may play a significant role in its characteristic manifestations.

To investigate these metabolic disruptions, targeted metabolomics offers a powerful tool. This approach allows for the precise quantification of specific metabolites within defined pathways^23–25^. In contrast to untargeted metabolomic approaches, which broadly screen for a wide range of metabolites, targeted metabolomics provides greater sensitivity and specificity, enabling the accurate identification of alterations in amino acid and fatty acid metabolism that may be linked to the clinical features of WS.

In this study, we conducted targeted metabolomic analysis on children with WS using liquid chromatography-tandem mass spectrometry (LC-MS/MS). Our objective was to identify alterations in amino acid and fatty acid metabolism, assess their potential implications for the clinical features of WS, and propose directions for future therapeutic research.

## Results

### Study cohort

Children diagnosed with Williams Syndrome (WS) (*n* = 29) and healthy controls (*n* = 32) were recruited for this study. There were no statistically significant differences between the groups in terms of age (*p* = 0.600) and gender distribution (*p* = 0.411) (Table 1).

**Table 1:** Baseline characteristics of the enrolled participants (*n* = 61).

	Control (*n* = 32)	WS (*n* = 29)	*p* value
Age (years)^**a**^	4.9 ± 1.9	4.6 ± 2.1	0.6
Gender, n (%)^**b**^			0.411
Female	10 (31.25)	12 (41.38)	
Male	22 (68.75)	17 (58.62)	

a. T-test, b. Chi-square test

The clinical phenotypes of WS patients are comprehensively outlined in Table 2. Notably, developmental delay was universally observed among all WS participants. The predominant phenotypes in WS patients included congenital heart disease (96.6%), with specific manifestations such as supravalvular aortic stenosis (SVAS, 93.1%), peripheral pulmonic stenosis (PPS, 51.7%), supravalvular pulmonary stenosis (SVPS, 27.6%), atrial septal defect (ASD, 6.9%), ventricular septal defect (VSD, 6.9%). It’s worth noting that inguinal hernia exhibited a higher prevalence in males than females (31.0% VS. 0.00%, *p* = 0.002), while other anomalies demonstrated varying incidence rates with no statistically significant gender differences. No significant differences were observed in other basic information between genders (Table 3).

**Table 2:** Clinical phenotypes in children with WS (*n* = 29).

Clinical phenotypes	Incidence	%	Female	%	Male	%	*p* value
Developmental delay	29/29	100	12/12	100	17/17	100	a.
Congenital heart disease	28/29	96.6	11/12	91.67	17/17	100	0.226
SVAS	27/29	93.1	10/12	83.33	17/17	100	0.081
PPS	15/29	51.7	6/12	50.00	9/17	52.9	0.876
SVPS	8/29	27.6	4/12	33.33	4/17	23.5	0.561
ASD	2/29	6.9	1/12	8.33	1/17	5.9	0.798
VSD	2/29	6.9	1/12	8.33	1/17	5.9	0.798
Subclinical hypothyroidism	11/29	37.9	5/12	41.67	6/17	35.3	0.728
Inguinal hernia	9/29	31.0	0/12	0	9/17	52.9	0.002**
Thyroid Dysgenesis	7/29	24.1	4/12	33.33	3/17	17.6	0.342
Hypercalciuria	3/29	10.3	1/12	8.33	2/17	11.8	0.765
Renal anomalies	2/29	6.9	1/12	8.33	1/17	5.9	0.305
Hypercalcemia	1/29	3.4	1/12	8.33	0/17	0	0.226
Hypothyroidism	2/29	6.9	2/12	16.67	0/17	0	0.081

a. No statistics are computed because developmental delay is a constant. **P＜0.01

**Table 3:** Comparison of basic information between male and female group in children with WS.

	Female(*n* = 12)	Male(*n* = 17)	*p* value ^a^
GA(w)	39.1 ± 1.7	39.0 ± 1.9	0.942
Age(y)	4.5 ± 2.1	4.3 ± 2.2	0.792
BW (kg)	2.7 ± 0.4	2.7 ± 0.5	0.798
Weight (kg)	14.5 ± 5.2	14.8 ± 4.5	0.880
BL (cm)	48.1 ± 2.6	46.9 ± 5.0	0.477
Height(cm)	98.5 ± 15.6	98.9 ± 15.5	0.935

a. T-test

## Metabolomic Profiling revealed significant alterations in WS

**Fig. 1:**
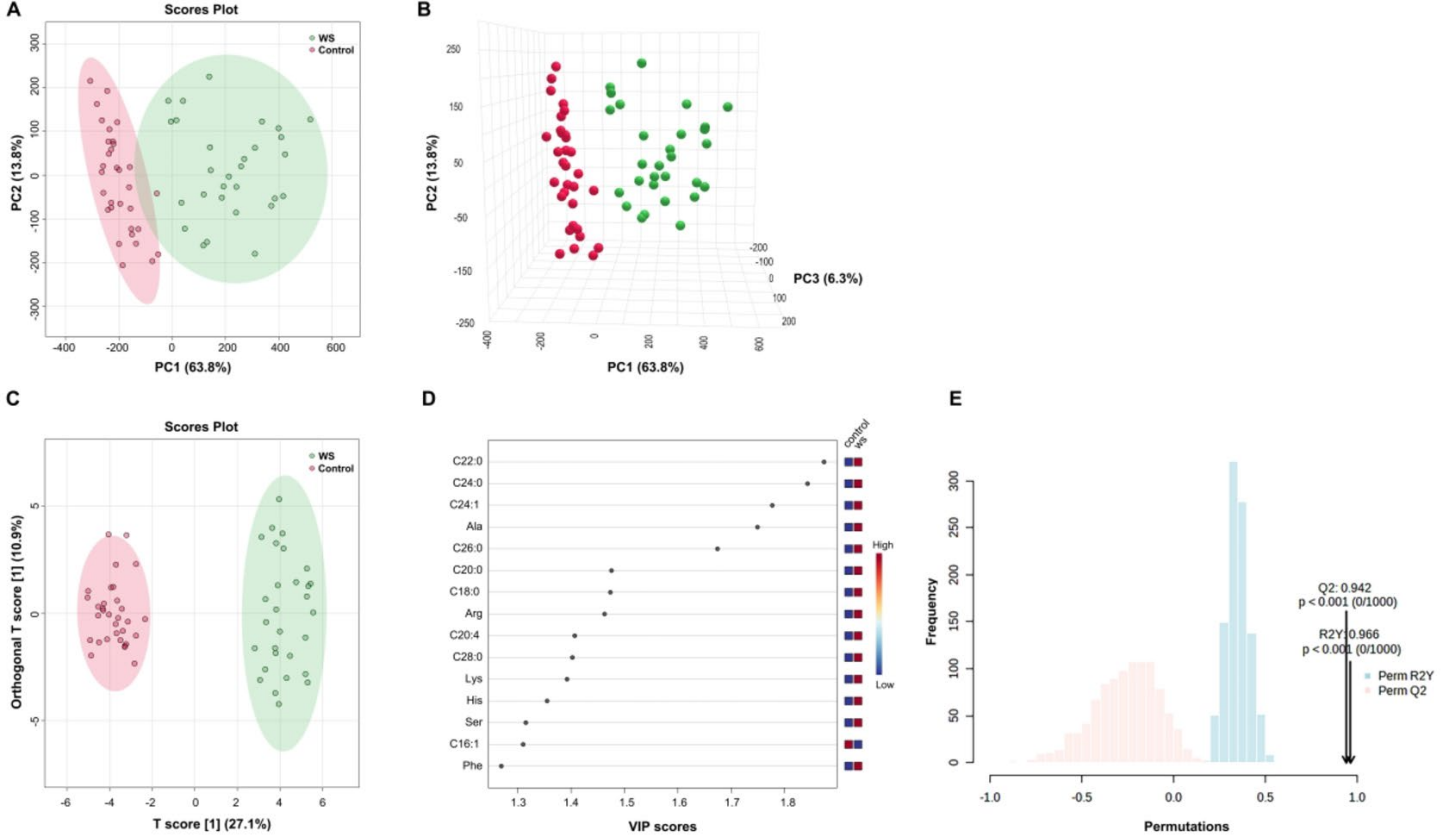
Principal Component Analysis (PCA), Three-Dimensional PCA (3D PCA), and Orthogonal Partial Least Squares Discriminant Analysis (OPLS-DA) were employed to assess amino acid and fatty acid metabolites, revealing a clear separation between the WS and control groups. (**a**) PCA score plots. (**b**) 3D PCA score plots. (**c**) OPLS-DA score plots. (**d**) Variable Importance in Projection (VIP) scores of OPLS-DA. (**e**) Results of Permutation Tests (*n* = 1000) Confirming the robustness and validity of the OPLS-DA Model.

The score plots derived from principal component analysis (PCA), three-dimensional PCA (3D PCA), and orthogonal partial least squares discriminant analysis (OPLS-DA) revealed a clear separation between the WS and control groups (Fig. 1a-c). Furthermore, the OPLS-DA model facilitated the assessment of metabolite contributions through variable importance in projection (VIP) scores (Fig. 1d and Supplementary Table S1). Permutation tests (*n* = 1000) with R2Y = 0.966(*p* < 0.001) and Q2 = 0.942(*p* < 0.001), affirmed the robustness and validity of OPLS-DA model (Fig. 1e).

**Fig. 2:**
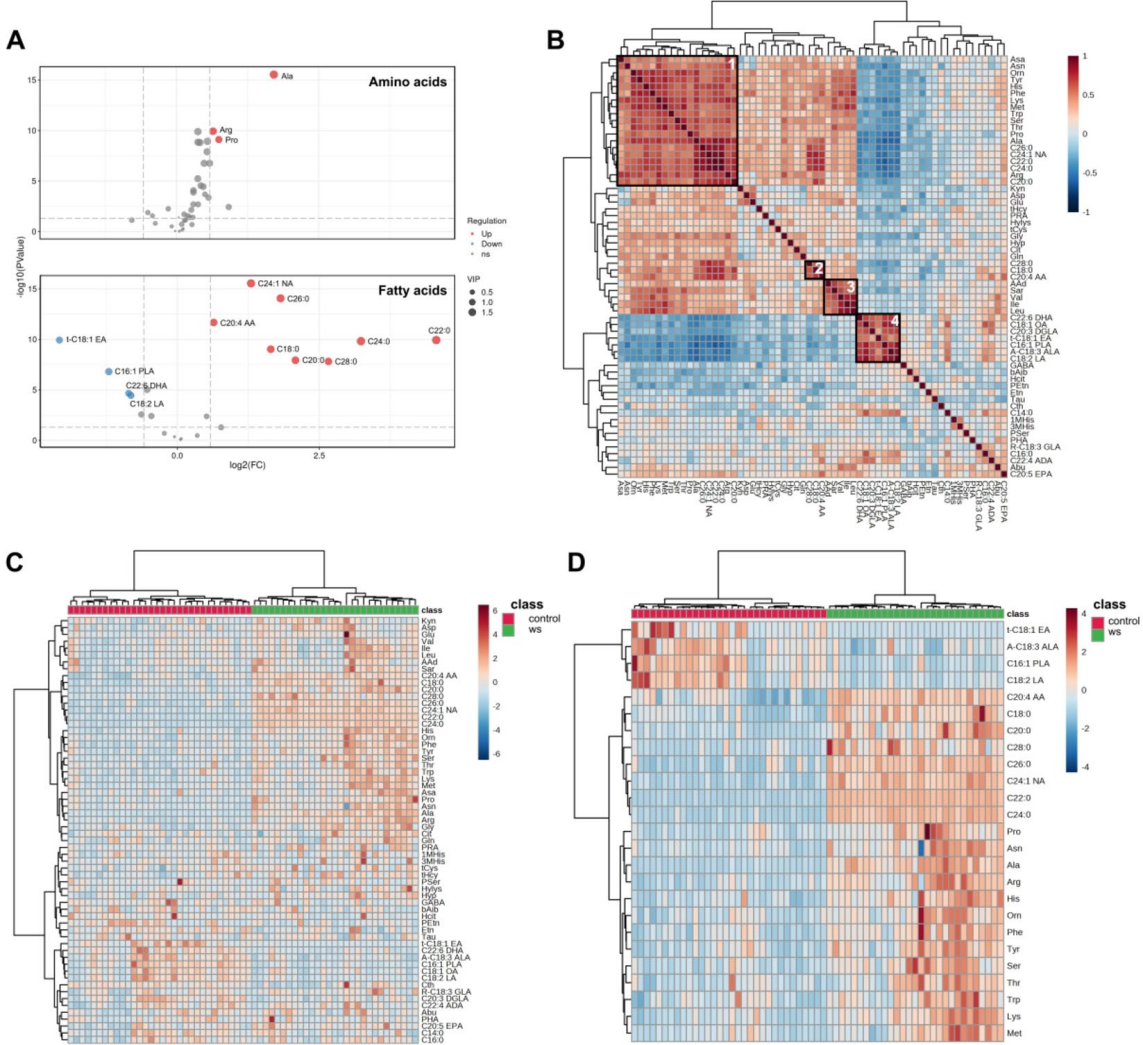
Comparative Analysis of Metabolic Profiles Between Williams Syndrome (WS) and Healthy Control Groups. (**a**) The volcano plot analysis identified significant changes in 15 metabolites. At the top, 3 out of the 40 detected amino acids were significantly upregulated. At the bottom, of the 22 detected organic acids, 4 were significantly downregulated and 8 were significantly upregulated. Differential metabolites were defined as those with a fold change > 1.5 in WS compared to healthy controls. A threshold of VIP > 1.0 and FDR < 0.05 was used to distinguish differential metabolites from non-significant ones. (**b**) Hierarchical clustering of Spearman’s rank correlation of change in metabolite levels. Clusters 1–4 were selected based on distinct correlation patterns among the features, as well as significant differences observed when compared to the control group. Red represents positive correlations and blue represents negative correlations. (**c**) Hierarchical clustering analysis demonstrates distinctive metabolic profiles between WS and healthy control groups. (d) The heatmap displays differences in the top 25 metabolites with the most significant changes between WS and healthy controls.

Among the 65 metabolites analyzed, anserine and carnosine were excluded from subsequent analyses due to their undetectable levels in multiple samples. A total of 15 distinct metabolites exhibited significant changes (FC > 1.5, FDR < 0.05, VIP > 1) (Fig. 2a and Supplementary Table S1). In WS, there was a general elevation of amino acids relative to the control, with notable increases in alanine (FC = 3.27), proline (FC = 1.67) and arginine (FC = 1.56). Additionally, 10 amino acids, including three aromatic amino acids (tyrosine, FC = 1.40; phenylalanine, FC = 1.30; tryptophan, FC = 1.30), showed relative fold enrichments ranging from 1.2 to 1.5 in WS (Supplementary Table S1).

Regarding fatty acids metabolism, long-chain saturated fatty acids (C18:0, C20:0, C22:0, C24:0, C28:0, C26:0) were significantly upregulated in WS, with C22:0 (FC = 23.49), C24:0 (FC = 9.39), and C28:0 (FC = 6.31) emerging as the top three upregulated LC-SFAs. Conversely, long-chain unsaturated fatty acids (C18:2 LA, C22:6 DHA, C16:1 PLA, and t-C18:1 EA) exhibited marked downregulation. Additionally, C18:3 ALA, one type of omega-3 PUFA, showed a downregulation in WS, although the fold change did not reach 1.5 (FC = 0.69, Supplementary Table**S1**). Interestingly, two long-chain unsaturated fatty acids (C24:1 NA and C20:4 AA) were upregulated in WS, displaying a similar expression pattern to LC-SFAs.

Subclinical hypothyroidism (SCH) is characterized by elevated thyroid-stimulating hormone (TSH) levels with normal free thyroxine (T4) levels. It has been linked to various metabolic disorders and cardiovascular risks^26^. Among the 29 WS patients included in our study, 11 exhibited subclinical hypothyroidism. We investigated the relationship between subclinical hypothyroidism and amino acid/fatty acid metabolites. Nine differential metabolites reached statistical significance, with only two exhibiting changes greater than 1.5-fold: argininosuccinic acid and C18:1 OA. After adjusting for *p*-values using the false discovery rate (FDR), only glutamine remained statistically significant, with a fold change of 1.18 (Table 4). Subsequent correlation analysis between glutamine and TSH, T4 showed no significant associations (*r* = 0.262, *p* = 0.170; *r*=−0.160, *p*= 0.408). A plasma metabolomics study on clinical hypothyroidism (CH) and subclinical hypothyroidism (SCH) indicated that, compared to the control group, subjects with SCH and CH exhibited a significant increase in L-arginine and a decrease in glycine. Levels of D-aspartic acid, indole-3-acetaldehyde, and indole-3-ethanol were significantly elevated in SCH but not in CH^27^. Additionally, a study on the relationship between essential micronutrients and thyroid function in a healthy population revealed a negative correlation between glutamine and T4 (*r* = − 0.1955, *p* < 0.0001)^28^. The differential metabolites identified in our stratified analysis of SCH in WS differ from those in the aforementioned studies. These discrepancies may be due to differences in the study populations, methodologies, and sample sizes.

**Table 4:** Metabolite Differences between subclinical hypothyroidism (SCH) and Control in WS.

Metabolite		FC	log2(FC)	*p* value	FDR
**AA**	Asa	1.60	0.67676	0.035581a	0.31084
	Cit	1.19	0.25594	0.026211a	0.31084
	**Gln**	**1.18**	**0.23825**	**0.00063254a**	0.037953*
	Asp	0.84	−0.24555	0.017633a	0.31084
	Kyn	0.84	−0.25963	0.036264a	0.31084
	Glu	0.67	−0.56961	0.0050999b	0.102
**FA**	C18:1 OA	1.67	0.74317	0.0021983b	0.065948
	C14 MA	1.43	0.51882	0.027513b	0.23582
	C16:1 PLA	1.30	0.38325	0.03503b	0.26272
a. T-test, b. Mann-Whitney U tests, *0.01＜P＜0.05					

## Correlation analysis

Correlation analysis was performed to explore the relationships among all features (Fig. 2b), revealing four distinct clusters. In Cluster 1, a prevalent upregulation of metabolites was observed, encompassing seven distinct metabolites with fold changes exceeding 1.5 (alanine, proline, arginine, C20:0, C22:0, C24:0, and C26:0), nervonic acid (C24:1), and ten amino acids with fold changes ranging from 1.2 to 1.5. Cluster 2 comprised three distinct metabolites with fold changes exceeding 1.5 (C28:0, C18:0, and C20:4). Cluster 3 included three branched-chain amino acids (leucine, isoleucine, and valine), along with sarcosine, which has been associated with potential benefits for patients with schizophrenia as an adjuvant treatment^29–31^, and α-aminoadipic acid, identified as a biomarker for diabetes risk^32^. Cluster 4 consisted of four distinct metabolites with fold changes exceeding 1.5 (C18:2 LA, C22:6 DHA, C16:1 PLA, and t-C18:1 EA), alongside C18:3 ALA and C20:3 DGLA. Metabolites within each cluster exhibited a notable positive correlation. Furthermore, metabolites in Cluster 1 demonstrated a significant positive correlation with those in Clusters 2 and 3, while metabolites in Cluster 4 exhibited a significant negative correlation with metabolites in Clusters 1, 2, and 3. This clustering analysis offers a comprehensive perspective on the interrelationships among metabolites, providing insights into potential metabolic pathways and interactions.

## Cluster analysis

Subsequently, we performed a comprehensive hierarchical clustering analysis of the metabolites, unveiling significant discrepancies in both the concentrations and patterns of various metabolites between individuals with WS and the healthy control group (Fig. 2c**)**. Focusing specifically on the top 25 metabolites displaying the most notable alterations, matrices observed in both the WS and control groups were consistent with clusters derived from correlation analysis, i.e. metabolites within Cluster 1 exhibited an upregulated expression pattern in WS, while those in Cluster 4 displayed a downregulated expression trend (Fig. 2d).

## Metabolomics Pathway Analysis (MetPA)

**Fig. 3:**
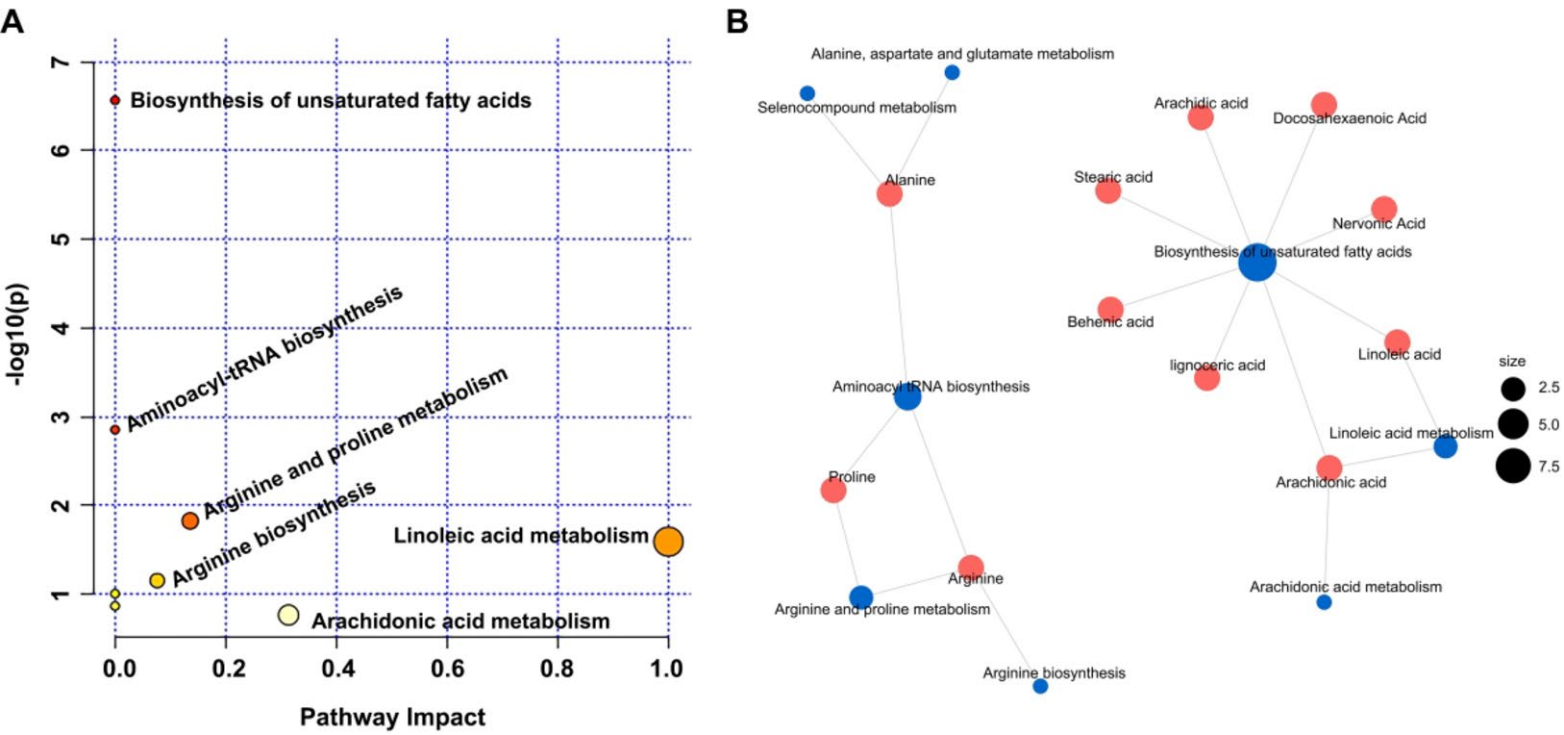
Metabolomics pathway analysis (MetPA). (**a**) MetPA bubble plots (**b**) Network view of MetPA.

Metabolomics Pathway Analysis (MetPA) was conducted using significantly altered metabolites, and the KEGG database was selected for this analysis (Fig. 3). Eight distinct metabolic pathways were identified, with four pathways reaching statistical significance (*p* < 0.05), including the biosynthesis of unsaturated fatty acids, biosynthesis of aminoacyl-tRNA, metabolism of arginine and proline, and metabolism of linoleic acid. Linoleic acid metabolism exhibits the highest pathway impact value.

## Discussion

Williams syndrome (WS) is a multisystem neurodevelopmental disorder affecting the cardiovascular, central nervous, gastrointestinal, and endocrine systems. While some gene-phenotype relationships have been elucidated, such as the role of the *ELN* gene in vascular and connective tissue features and the transcription factor genes *GTF2I* and *GTF2IRD1* with intellectual abilities, substantial phenotypic variability exists within WS. The mechanisms behind this variation remain largely unknown, necessitating further research to understand the factors influencing clinical outcomes. In this study, we used LC-MS/MS to investigate amino acids and fatty acids profiles in children with WS. To our knowledge, this is the first study characterizing fatty acid and amino acid metabolism in WS through targeted metabolomics.

WS exhibits a distinctive neurodevelopmental phenotype characterized by developmental delays, mild intellectual disability, and deficits in visuospatial construction. Individuals with WS often show hypersocial behaviors, attention problems (consistent with ADHD), social functioning issues, and anxiety, resembling features of neurodevelopmental disorders such as ADHD and autism spectrum disorder (ASD), which are commonly associated with inflammatory pathology. Maintaining a balance between omega-6 and omega-3 PUFAs is critical for optimal brain function, as these PUFAs have distinct roles. Omega-3 PUFAs (EPA, DPA, and DHA) are recognized for their anti-inflammatory properties, while omega-6 PUFAs (ARA) are associated with pro-inflammatory processes^33–37^. The antagonistic relationship between DHA and ARA suggests that DHA’s modulation of ARA metabolism may mitigate symptoms of such disorders. Lower blood levels of omega-3 PUFAs, particularly DHA, in children with ADHD and ASD have been noted^38–40^. Our study confirmed dysregulation in WS, with upregulated pro-inflammatory arachidonic acid (ARA) and downregulated anti-inflammatory docosahexaenoic acid (DHA). This finding aligns with existing reports, suggesting a potential role for the DHA-ARA antagonistic interaction in WS neurodevelopmental phenotypes. This enhances our understanding and provides potential new targets for future management and intervention. Dietary supplementation with omega-3 PUFAs shows promise for rebalancing the omega-6 to omega-3 ratio in WS, potentially alleviating inflammation and improving neurodevelopmental outcomes. Omega-3 PUFAs have emerged as a promising therapeutic option due to their safety, tolerability, and potential cognitive benefits^41–43^. Despite these promising findings, conflicting results have been reported^44–46^, possibly due to variations in study populations. Further research is crucial to clarify the specific impact of omega-3 PUFAs on cognitive function across diverse age groups and health conditions.

Chierico et al. conducted an exploratory analysis of the gut microbiota in individuals with Williams syndrome (WS), revealing a dysbiosis characterized by an increased abundance of pro-inflammatory bacteria compared to controls^47^. This dysbiosis was associated with reduced production of short-chain fatty acids (SCFAs), which are known for their anti-inflammatory properties, indicating an inflammatory state at the intestinal level in WS^48^. Our study supports these findings by demonstrating an upregulation of pro-inflammatory arachidonic acid (ARA) and a downregulation of anti-inflammatory docosahexaenoic acid (DHA) in individuals with WS compared to controls. Additionally, we observed a significant downregulation of palmitoleic acid, an omega-7 monounsaturated fatty acid (FC = 0.43, Fig. 2a, and **Supplementary Table ****S1**), which has been shown in previous research to exert anti-inflammatory and anti-colitis effects by modulating gut microbiota. Collectively, these findings suggest a pro-inflammatory shift in WS, highlighting the role of both microbial and metabolic dysregulation in contributing to an inflammatory environment. Addressing these dysregulations holds promise for improving health outcomes and managing comorbid conditions associated with WS.

Supravalvular aortic stenosis (SVAS), common in WS, is mainly caused by mutations in the elastin (*ELN)*gene. Although immunity was not previously linked to SVAS risk, Parrish et al. found a connection between SVAS severity and adaptive immune system genes^49^. Introducing the *Rag1*^*−/−*^ mutation^50^, which impairs adaptive immunity, into a mouse model with aortic stenosis and high blood pressure improved aortic size and reduced blood pressure^49^. Further analysis identified key genes involved in B-cell activation and proliferation as related to WS traits^51^. These findings highlight the potential of targeting the adaptive immune system to improve WS prognosis. Omega-3 polyunsaturated fatty acids (PUFAs), known for modulating both innate and adaptive immune responses^52–56^, are markedly down-regulated in WS. Exploring the complex interactions between unsaturated fatty acids, particularly DHA and ARA, and their roles in inflammation could provide insights into their impact on WS neurodevelopmental phenotypes and SVAS.

Our study highlights an interplay between amino acid and fatty acid metabolism in WS, which may provide insights into potential metabolic alterations linked to diabetes risk. Adults with WS show increased susceptibility to diabetes, with a lower incidence in children and a significant rise during adolescence^57,58^, suggesting a developmental component to this susceptibility. Alterations in amino acid and fatty acid metabolism add further complexity to this relationship. While existing literature associates elevated levels of alanine, aromatic amino acids (tyrosine, phenylalanine, tryptophan), and branched-chain amino acids (BCAA) with an increased risk of type 2 diabetes (T2D)^59–63^, our study reveals a distinct metabolic profile in WS. Consistent with prior reports, alanine and aromatic amino acids (AAA) were upregulated in WS (Supplementary Table S1). However, the absence of significant differences in circulating BCAA levels between WS patients and controls warrants further investigation into the specific metabolic landscape of WS.

Exploration of fatty acid metabolism in WS reveals an unexpected trend. Despite research linking very-long-chain saturated fatty acids (VLSFAs) with a lower risk of diabetes^64–66^, WS patients show increased levels of VLSFAs, particularly 20:0, 22:0, and 24:0. This increase is coupled with a notable decrease in elaidic acid, a trans fatty acid associated with higher diabetes risk^67,68^. This paradoxical finding, which contrasts with the higher prevalence of diabetes during adolescence in the general population, highlights the complexity of WS metabolism. To elucidate these mechanisms, longitudinal studies tracking metabolic changes during diabetes development in WS patients are essential. Such research could lead to better biomarkers for managing and treating diabetes in WS.

WS patients typically face unique dietary challenges, including feeding difficulties during infancy, sensory sensitivities, and specific dietary recommendations due to cardiovascular and gastrointestinal issues. These challenges can significantly impact their metabolic profiles. Nutritional deficiencies resulting from feeding problems and selective eating can lead to imbalanced nutrient intake and alterations in metabolic biomarkers. Restricted dietary diversity may cause metabolic disturbances, exacerbating the health challenges associated with WS. Poor diet quality can also affect blood lipids, increasing the risk of cardiovascular disease and type 2 diabetes. Therefore, comprehensive nutritional assessments, personalized dietary plans, and targeted interventions are crucial for optimizing metabolic health and enhancing the overall quality of life for WS patients. Our study highlights significant changes in the amino acid and organic acid metabolic profiles of children with WS. These findings provide preliminary insights into how dietary interventions and nutritional support might improve the metabolic health and quality of life for WS patients. However, further research is needed to explore the long-term impact of these dietary issues and to identify effective interventions for improving dietary intake and metabolic outcomes. Longitudinal studies tracking dietary intake, growth parameters, and metabolic characteristics from childhood to adulthood will be crucial for understanding the long-term effects of these dietary challenges.

In summary, the significant changes in amino acid and fatty acid profiles align closely with the clinical phenotype of Williams syndrome. Omega-3 and omega-6 polyunsaturated fatty acids (PUFAs) may influence various phenotypes of WS through their effects on inflammation and immunity, making them potential novel targets for future management and intervention. However, this study has several limitations. The small sample sizes limit the generalizability of the findings, and the metabolic profiles in WS individuals may vary widely. Further research is needed to investigate potential subtypes or subtle metabolic dysregulations. Moreover, the current analysis was confined to amino acids and fatty acids, leaving other metabolites unexamined. Future studies employing untargeted metabolomics could facilitate the identification of additional biomarkers associated with WS, which could subsequently be validated through targeted approaches. This comprehensive strategy would offer a deeper understanding of the syndrome’s metabolic alterations.

## Methods

### Subject Selection

This study included 29 patients with WS diagnosed (Age, 4.6 ± 2.1 years; Gender, 17 males, 12 females) and 32 healthy individuals (Age, 4.9 ± 1.9 years; Gender, 22 males, 10 females) in the Children’s Hospital affiliated with Zhejiang University School of medicine (Table 1). The clinical diagnosis of Williams syndrome in our study utilized chromosomal microarray (CMA) and multiplex ligation-dependent probe amplification (MLPA) to detect microdeletions at the 7q11.23 locus.

### Sample Preparation

Blood samples were collected from all participants after an overnight fast to ensure consistency and minimize variability in metabolic markers. Venipuncture was performed by trained phlebotomists using standard procedures, with samples collected in gold-top serum-separating tubes. To maintain sample quality, all samples were processed within two hours of collection.

### LC-MS/MS Targeted Metabolomics

The detection of 43 amino acids and 22 free fatty acids was conducted on the API 4500 liquid chromatography-tandem mass spectrometry (LC-MS/MS) system (Triple Quad™ 4500MD, AB Sciex, MA, USA).

### Amino Acids Detection

Extract 50 µL of serum into a 1.5 mL centrifuge tube. Add 200 µL of methanol containing internal standards and 20 mg/mL dithiothreitol (DTT) with 0.1% formic acid (FA). Vortex the mixture thoroughly, and centrifuge it at 14,000 rpm at 4 °C for 10 min. Transfer 200 µL of the supernatant to a 2 mL centrifuge tube and evaporate it under nitrogen gas at 40 °C. Reconstitute the dried sample in 100 µL of a mobile phase composed of solvent A and solvent B in a 1:2.5 ratio (70% acetonitrile). After thorough vortex mixing for 3 min, centrifuge the mixture again at 14,000 rpm at 4 °C for 10 min. Filter the supernatant through a 0.2 μm membrane filter to obtain the test sample. Transfer the filtered sample to an injection vial for subsequent targeted metabolomics analysis.

The extracted metabolites were subjected to chromatographic analysis using an ACQUITY UPLC BEH Amide Column (1.7 μm, 2.1 mm x 100 mm, Waters Ltd, Elstree, UK). Mobile phase A: 0.1% formic acid, 10mM ammonium formate, and acetonitrile/water (20:80). Mobile phase B: 0.1% formic acid and 10mM ammonium formate, and acetonitrile/water (10:90). The column temperature was maintained at 45℃, with an injection volume of 3 µl a flow rate of 400 µl/min. Electrospray ionization (ESI) in positive ion mode was employed, using Scheduled MRM™ (sMRM) mode to obtain high-quality full-scan sub-ion spectra. The mass spectrometry conditions included an ion source temperature of 550℃ and an electrospray voltage of 5500 V. Optimal mass spectrometry acquisition conditions and corresponding multiple reaction monitoring (MRM) ion pairs for each amino acid were determined using individual standard solutions. Identification of the analytes was achieved based on the retention times **(**Supplementary Table S2**)** and characteristic ion pairs of each analyte and its internal standard.

### Fatty acid detection

Extract 50 µL of serum into a 1 mL 96-well plate. Add 200 µL of a precipitant (methanol: n-hexane, FA = 4:1, 0.05%) containing internal standards. Vortex the mixture thoroughly, then add 400 µL of n-hexane and mix well. Centrifuge the mixture at 3,000 rpm and 10 °C for 10 min. Using a multichannel pipette (with a tip rack), transfer the supernatant to a 400 µL 96-well plate and evaporate the solvent under a gentle stream of nitrogen. Reconstitute the residue in 100 µL of 85% methanol-water, mix thoroughly, and inject 3 µL of the reconstituted sample for analysis.

The extracted metabolites were subjected to chromatographic analysis using an ACQUITY UPLC BEH C18 Column (1.7 μm, 2.1 mm x 50 mm, Waters Ltd, Elstree, UK). Mobile phase A: 0.1% NH4OH, 10mM ammonium formate, and acetonitrile/water (10:90). Mobile phase B: 0.1% NH4OH, 10% IPA, 90% ACN. The column temperature was maintained at 35℃, with an injection volume of 3 µl a flow rate of 400 µl/min. Electrospray ionization (ESI) in negative ion mode was employed, using Scheduled MRM™ (sMRM) mode to obtain high-quality full-scan sub-ion spectra. The mass spectrometry conditions included an ion source temperature of 400℃ and an electrospray voltage of −4500 V. Optimal mass spectrometry acquisition conditions and corresponding multiple reaction monitoring (MRM) ion pairs for each fatty acid were determined using individual standard solutions. Identification of the analytes was achieved based on the retention times **(Supplementary Table ****S2****)** and characteristic ion pairs of each analyte and its internal standard.

To ensure reproducibility, quality control samples were included in each batch of analyses to monitor instrument performance and consistency in sample preparation. For each batch, three quality control products of different concentrations were tested simultaneously to ensure that the test values were within ± 2SD of the target value, and the coefficient of variation (CV) between batches was ≤ 10%. Internal standards were added to all samples to account for any variability in sample preparation and instrument performance. Detailed information on internal standards, working solutions, target analyte quantification, and calibration parameters is provided in Supplementary Table**S2**. Additionally, the representative chromatograms for each assay are presented in Supplementary Figure**S1**.

### Statistical analysis

MetaboAnalyst v5.0 was employed for the statistical analysis of targeted metabolomics data (https://www.metaboanalyst.ca/)^69^. The performance of the Orthogonal Partial Least Squares Discriminant Analysis (OPLS-DA) model was assessed based on model fitness (R2Y) and predictive power (Q2) values. Variable Importance in Projection (VIP) scores were utilized to evaluate the contributions of individual metabolites in the OPLS-DA model. Correlation analysis to assess the correlation between all features was conducted using the Pearson correlation coefficient (r) as a distance measure. Hierarchical clustering heatmaps, generated from the original datasets, were produced using Ward’s linkage for clustering and Euclidean distance measure. Metabolomics pathway analysis (MetPA) was performed using significantly altered metabolites, and the KEGG database was selected for this analysis.

Additionally, volcano plots and network view of MetPA were generated using the BioDeep Platform (https://www.biodeep.cn/home/tool). The criteria for defining these alterations are defined based on a fold change (FC) cutoff of 1.5, a false discovery rate (FDR) less than 0.05, and VIP values exceeding 1.

Univariate analysis was carried out using SPSS 26.0 for Mac (IBM, Armonk, NY, USA). The Shapiro-Wilk test was used to assess the normality of the data distribution. Depending on the data distribution, both parametric and non-parametric tests were applied. Specifically, t-tests were used for normally distributed data, and Mann-Whitney U tests were used for non-normally distributed data. To control the false discovery rate (FDR) in multiple comparisons, the Benjamini-Hochberg procedure was employed.

## Conclusion

In conclusion, we investigated into amino acids and fatty acids metabolism in children with Williams syndrome for the first time. This investigation successfully elucidates metabolic distinctions between WS patients and healthy controls. The comprehensive metabolic profiling of children with WS offers invaluable insights into the understanding of metabolic aberrations associated with this rare genetic condition. Further research endeavors are imperative to validate and expand upon these findings, potentially leading to the development of precise interventions and personalized therapeutic strategies for individuals affected by Williams syndrome.

## Electronic Supplementary Material

Below is the link to the electronic supplementary material.

Supplementary Material 1

Supplementary Material 2

## Data Availability

The data that support the findings of this study are available from the corresponding author upon reasonable request.

## Abbreviations

1MHis: 1-Methylhistidine
3MHis: 3-Methylhistidine
AAd: Aminoadipic acid
Abu: Alpha-aminonbutyric acid
Ala: Alanine
Arg: Arginine
Asa: Argininosuccinic acid
Asn: Asparagine
Asp: Aspartic acid
bAib: 3-Aminoisobutanoic acid
Cit: Citrulline
Cth: Cystathionine
tCys: Cysteine
Etn: Ethanolamine
GABA: Gamma-Aminobutyric acid
Gln: Glutamine
Glu: Glutamic acid
Gly: Glycine
Hcit: Homocitrulline
Hcy: Homocysteine
His: Histidine
Hylys: Hydroxylysine
Hyp: hydroxyproline
Ile: Isoleucine
Kyn: Kynurenine
Leu: Leucine
Lys: Lysine
Met: Methionine
Orn: Ornithine
PEtn: Phosphoethanolamine
Phe: Phenylalanine
Pro: Proline
PSer: Phosphoserine
Sar: Sarcosine
Ser: Serine
Tau: Taurine
Thr: Threonine
Trp: Tryptophan
Tyr: Tyrosine
Val: Valine
Ans: Anserine
Car: Carnosine
A-C18: 3:ALA Alpha-Linolenic acid
C14: 0:Myristic acid
C16: 0:Palmitic acid
C16: 1 PLA: Palmitoleic acid
C18: 0:Stearic acid
C18: 1 OA: Oleic acid
C18: 2 LA: Linoleic acid
C20: 0:Arachidic acid
C20: 3:DGLA cis-8,11,14-Eicosatrienoic acid
C20: 4 AA: Arachidonic acid
C20: 5 EPA: Eicosapentaenoic Acid
C22: 0:Behenic acid
C22: 4 ADA: Docosatetraenoic acid
C22: 6 DHA: Docosahexaenoic Acid
C24: 0:lignoceric acid
C24: 1 NA: Nervonic Acid
C26: 0:Hexacosanoic acid
C28: 0:Octacosanoic acid
PHA: Phytanic acid
PRA: Pristanic acid
R-C18: 3 GLA: Gamma linolenic Acid
t-C18: 1 EA: Elaidic Acid
