# Supplementary figures and images for: Metabolic profiling reveals altered amino acid and fatty acid metabolism in children with Williams Syndrome
Source: Sci Rep. 2024 Dec 28;14:31467. doi: 10.1038/s41598-024-83146-4 (PMC11682280; doi:10.1038/s41598-024-83146-4)

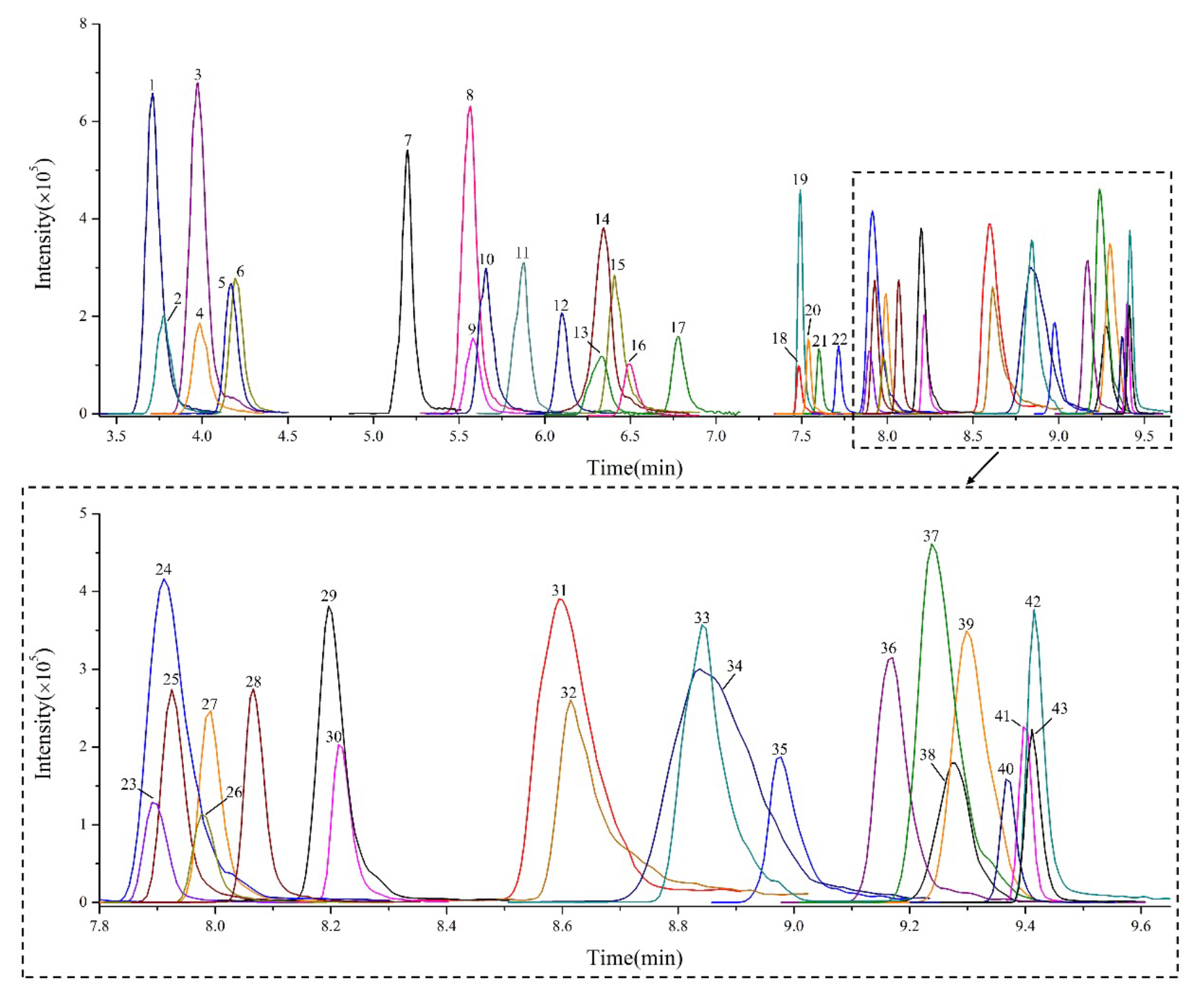


**Supplementary Figure S1. Representative chromatogram of assay.**

Supplement: Supplementary file 2 — Supplementary Material 2 [file 41598_2024_83146_MOESM2_ESM.docx]
